# Mobile sailing robot for automatic estimation of fish density and monitoring water quality

**DOI:** 10.1186/1475-925X-12-60

**Published:** 2013-07-01

**Authors:** Robert Koprowski, Zygmunt Wróbel, Agnieszka Kleszcz, Sławomir Wilczyński, Andrzej Woźnica, Bartosz Łozowski, Maciej Pilarczyk, Jerzy Karczewski, Paweł Migula

**Affiliations:** 1 Department of Computer Biomedical Systems, Institute of Computer Science, University of Silesia, Będzińska 39, 41-200, Sosnowiec, Poland; 2 Faculty of Mining Surveying and Environmental Engineering, AGH University of Science and Technology, Krakow, Poland; 3 Department of Cosmetology, Katowice School of Economics, ul. Harcerzy-Wrzesnia 3, 40-659, Katowice, Poland; 4 Faculty of Biology and Environmental Protection, University of Silesia, 40-032, Katowice, Poland; 5 Polish Academy of Sciences in Gołysz, Kalinowa 2, Zaborze, 43-520, Chybie, Poland

**Keywords:** Robot, Drinking water, Fish, Monitoring, Water quality

## Abstract

**Introduction:**

The paper presents the methodology and the algorithm developed to analyze sonar images focused on fish detection in small water bodies and measurement of their parameters: volume, depth and the GPS location. The final results are stored in a table and can be exported to any numerical environment for further analysis.

**Material and method:**

The measurement method for estimating the number of fish using the automatic robot is based on a sequential calculation of the number of occurrences of fish on the set trajectory. The data analysis from the sonar concerned automatic recognition of fish using the methods of image analysis and processing.

**Results:**

Image analysis algorithm, a mobile robot together with its control in the 2.4 GHz band and full cryptographic communication with the data archiving station was developed as part of this study. For the three model fish ponds where verification of fish catches was carried out (548, 171 and 226 individuals), the measurement error for the described method was not exceeded 8%.

**Summary:**

Created robot together with the developed software has features for remote work also in the variety of harsh weather and environmental conditions, is fully automated and can be remotely controlled using Internet. Designed system enables fish spatial location (GPS coordinates and the depth). The purpose of the robot is a non-invasive measurement of the number of fish in water reservoirs and a measurement of the quality of drinking water consumed by humans, especially in situations where local sources of pollution could have a significant impact on the quality of water collected for water treatment for people and when getting to these places is difficult. The systematically used robot equipped with the appropriate sensors, can be part of early warning system against the pollution of water used by humans (drinking water, natural swimming pools) which can be dangerous for their health.

## Introduction

Distribution of wild fish is rarely homogeneous since it is determined by such parameters as e.g.: physical and chemical conditions of the water and food availability [1]. Temperature affects species composition or cause individuals to seek thermal refugees [2]. Alterations in the dynamics and growth rate of fish populations and their structure may lead to a series of adverse changes in the water bodies, such as decreased water quality, or losses in revenues from the harvesting fish. Changes of fish behavior during discharge disturbances might have important management implications. Floods can reduce fish abundance, alter community structure or disturb natural fish displacements [3]. Proper environmental risk assessment of small inland water reservoirs or aquaculture fish ponds needs fast, cheap and effective tools useful in monitoring fluctuations of the population dynamics and density of fish throughout the whole season. Fishery stakeholders expect also the exact data to calculate turnover losses due to cormorants and other carnivorous birds. This means that techniques that can negatively influence fish condition and behavior, such as electrofishing, or others that may lead to overfishing, should not be recommended.

Sonars as the tools in quantitative assessment have been for many years successfully applied in the sea fisheries and in monitoring large inland water bodies. They are used in tracking the movement of large shoals of fish in the sea fisheries and also in recording the fishes migrating to their spawning grounds. Most commercially available sonars enable identification and determination of fishes. Many methods of the image analysis for sonars [4-8] or texture analysis for the object detection are recently available [9-12]. Usually sonars have built-in electronics, a display and a cable connected ultrasonic head. They are often equipped with a wireless, sailing head. Some sonars are tracking the number and behavior of fishes inhabiting a given water body. Sonars, despite recording the head frequency, are ready to transfer the data, track the trajectory and read the coordinates of the Global Positioning System (GPS) from the satellites. Many available models (e.g., Lowrance, Humminbird, Garmin, Eagle, Didson) support the transfer and collection of the data. Currently used sonars, depending on the used head, ensure the operation in the range of 200 kHz and 43 kHz. They detect the presence of fishes but their number could not be estimated.

At present, a reliable, reproducible and inexpensive method of estimation the number and density of freely swimming fishes in stillwater reservoirs is not available. By now, some drawbacks rather discourage the use of sonars to carry out such analysis. These include variability of fish species, their shape, behavior or age related differences and effects of climatic conditions. Net fishing or electrofishing may operate only in small areas. Moreover, net fishing contribute to high mortality of some fish species, and further problems with utilization of the carcasses.

In the aquaculture the initial number of the fish in the breeding ponds and their approximate age are generally known. During the season their number is difficult to estimate. Mewchanistic techniques used for their estimation might also affect fish behavior (Heisenberg uncertainty principle). The placement of any measuring device, irrespective of its size, and even appearance of its shade, may result in a variety of unpredictable swimming responses of fish. The acoustic analysis showed that 76% of the fish in the trawling area were captured and 24% swam out of it. The shadow of the boat was the primary reason for this situation while, other factors have a minor impact [13].

In summary, the problem to be solved is a reliable estimation of the fish number in reservoirs, using efficient, fast, cheap and readily available tools for this purpose. A key objective of this study was to develop an effective quantitative method to assess the number and density of the fish population with simulteneaously recorded various physicochemical environmental factors. The low-cost, remote-controlled boat model (robot) was developed, equipped with the sonar and/or other probes measuring physicochemical parameters in the water body. Through scanning the entire reservoir, the data necessary to estimate the number of fish were tested and verified in various environmental conditions.

Variability of many parameters such as changes of the depth of the analyzed reservoir, the angle of the sonar head cone, the differences between fish species or the depth of their foraging were taken into account. The tool constructed for estimation of the fish number is based on the data collected from a cheap sonar module mounted on a sailing robot. The sonar covering the set trajectory by the robot is supported with the module recording the time-space location of sonar images and physicochemical parameters of the water. This paper is given a detailed description of the developed functionality of the robot and the types of designing modules used in the pilot studies.

## The tools

The method of estimating the number of fish was based on three elements:

• a mobile sailing robot,

• the software for the wireless communication between the robot and the station,

• the calibration of the measurement technique.

**Figure 1:**
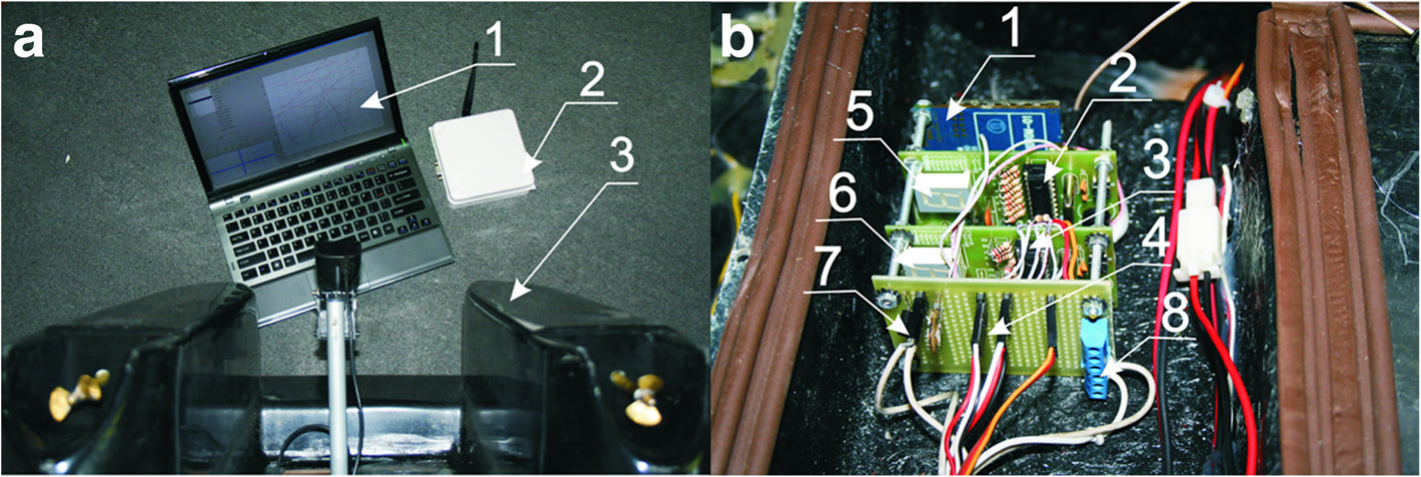
**General view and units of the robot microprocessor system. (a)** General view of the system (1– Laptop with the software designed to control and archive wirelessly transmitted data, 2– 2.4 GHz transmitter, 3– robot with a visible measuring head); **(b)** View of the central unit of the robot microprocessor system (1– serial transmitter module, 2,3– microprocessors, 4– connection of drive motor controllers, 5*,6*– segment displays, 7– connection of GPS with NMEA serial transmission, 8– 12V system power supply).

The mobile sailing robot (Figure 1) meets the following assumptions:

• control and data transmission in full duplex mode with encrypted transmission - communication module Aerocomm AC 4490LR-1000M-01,

• a catamaran type, due to greater stability of sailing in comparison with a classic boat,

• a wireless transmission of the GPS data, water temperature, compass coordinates, the number of available satellites, battery charging level, date retrieved from the satellite time, sailing speed,

• local data archiving from the Lowrance sonar unit directly in the boat (22 GB memory) and the possibility of their remote transmission,

• the range up to 10 km in an open water reservoir,

• 2.4 GHz wireless bandwidth, data transmission rate in full duplex mode at 57 kB/s,

• sounding an alarm (105 dB) in case of a sudden loss of communication or undue displacement,

• the robot control using a 51 microprocessor family.

The software meets the following assumptions:

• full communication with the robot: setting of all the parameters and visualization of the received data,

• archiving of data received from the robot,

• remote control in manual and automatic mode according to the set trajectory,

• automatic estimation of the number of fish in the reservoir,

• working in the Windows operating system.

The robot with particular emphasis on the control systems and the deployment of the sonar head is shown in Figure 1. The main part of the constructed robot – Figure 1 (a) is a catamaran-type vessel 0.70 m long, 0.53 m wide, and 0.30 m high with 6 kg displacement and weight below 15 kg. The robot is equipped with driving motors, speed recorders controlled with the duty ratio of the signal, a microprocessor-based control system, a sonar module, a module of water chemical parameter sensors and a wireless module with antenna. The software communicates with the system of wireless transmitter and receiver powered from a separate battery unit via USB. The transmitter operation is also possible from a computer by the USB port. Due to limited current intensity (500 mA), the communication range of the robot is limited to 1 km. Communication takes place at a cryptographically secured rate of 57 kB/s. The GPS coordinates and the trajectory of the movements are read and displayed on the screen. The application (station), allows with the mouse to select the area of the robot movements. Consecutive zigzag movements may cover the entire reservoir. In case of any problem with sticking to the route set (strong wind or other obstacles), the warning is displayed on the application screen. Switching to manual control is also possible, but only if the robot is in sight. In practice, manual control with no eye contact with the robot is difficult, especially at small distances when quick changes of the direction are not yet visible in changes of the GPS coordinates. The application also shows the read data related to the compass, speed of movement, the number of reading satellites, the battery level and selected physical-chemical parameters of the water. The data are also stored in the XML format and are sent to the server as the FTP data stream. Despite cryptographic transmission the secure work of the robot is additionally protected. The data transmitted in the 2.4 GHz band have additional security systems such as: the ID system, MAC address, RF Channel Number, Interface Timeout and the Destination address. Properly configured modules of wireless transmission only allow internal communication between each other, and the broadcast is switched off. Despite cryptographic data transmission the MAC address of the tools is also verified. Moreover, steering commands sent from the base station to the robot are dynamically coded. Changes of the trajectory or physical robot interception are one of the largest threats of the proper technical work. Our protective system significantly reduces potent breaking of the transmission protocol of the robot and changes of its movement trajectory.

The drive and control systems of the robot are powered separately. This made possible to send location information even if the mobility is completely blocked. When the movement of the robot is correct and the battery voltage drops from 12 V to 9 V, the appropriate information is sent to the station. If the operator does not take any controlling action the robot turns back to the station on the autopilot.

## The method

### Methodological bases

**Figure 2:**
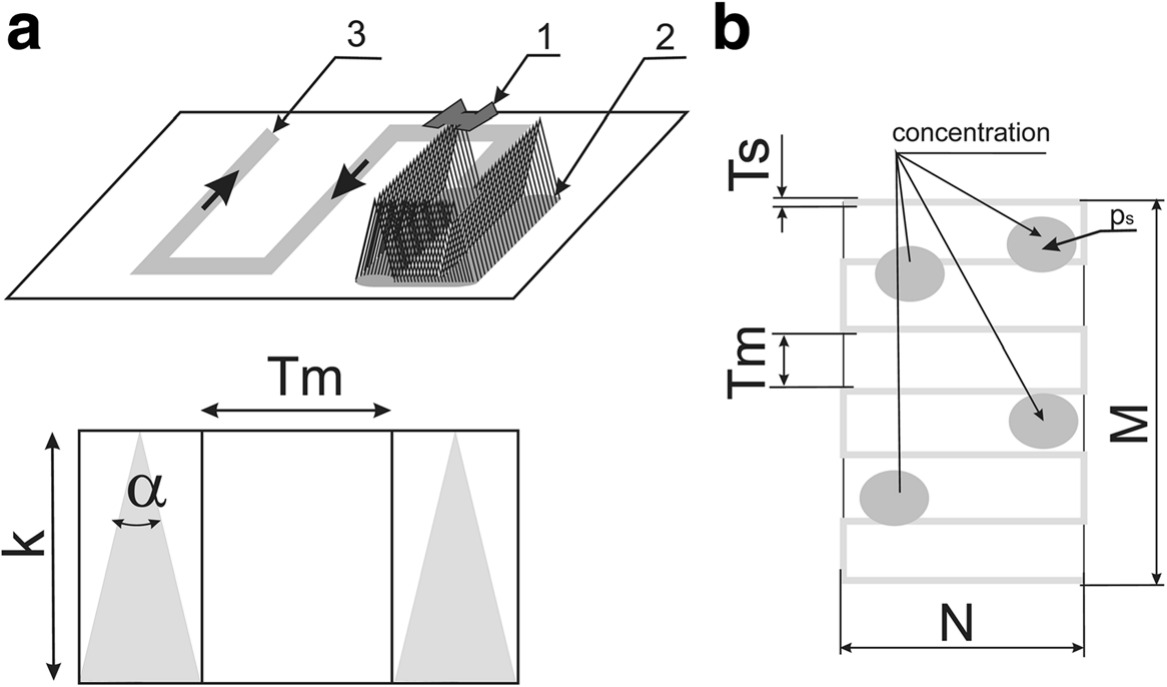
**The robot trajectory.** (**a**) The area covered by the sonar *Ts* with cone angle *α* and height *k* (1– robot, 2– sonar beam, 3– trajectory); (**b**) The way of marking the width of the sonar beam at the bottom (*Tm*- the distance between adjacent tracks, *p*_*s*_– a sample distribution of fish of the same surface).

The measurement method for estimating the number of fish using the described robot is based on a sequential calculation of the number of occurrences of fish on the set trajectory. The trajectory, set with the use of the developed software, takes the form of parallel lines arranged horizontally or vertically in the analyzed area (Figure 2). Figure 2(a) showed that the sonar is not providing a complete information on the whole volume of the reservoir which depends on the depth of the bottom surface. The volume of scanned volume depends on the ultrasound beam cone angle. Knowledge of the depth where individual fish are recorded, allows to make necessary corrections. To refine the analysis, it was assumed that the fish is present at a constant depth on the flat bottom. In further analysis this assumption will not be valid. Thus, the calculation of the measurement error related to the number of fish in the reservoir was based on the following assumptions:

• one constant depth of the fish location,

• fixed width of the sonar ultrasonic beam,

• each measurement is a mean value of 5000 simulations,

• rectangular trajectory of robot’s movement,

• random distribution of the fish with variable number of clusters – from 1 to 60.

The following variables were analyzed:

• variable number of fish clusters *s* ranging from 1 to 60,

• variable frequency of robot’s crossings comprising 10% to 100% of the analyzed reservoir surface *Tm/Ts*,

• variable cluster areas *p*_*s*_ covering 10% to 100% of the reservoir surface.

**Figure 3:**
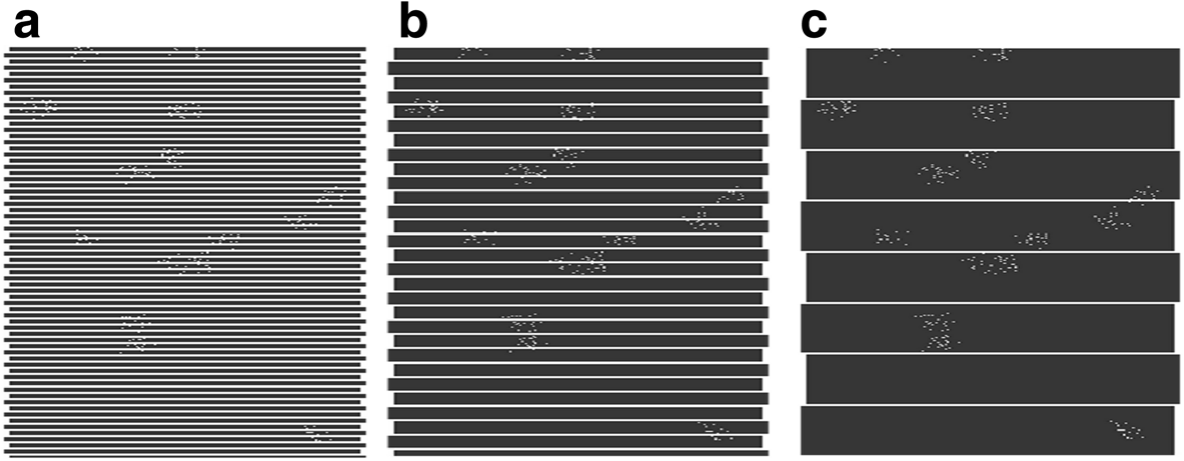
**Sample trajectories for the growing number of crossings representing: (a) 50%; (b) 30%; and (c) 10% of the total area.** Fish clusters are visible as randomly distributed white dots (set the number of clusters).

Sample trajectories for the robot movements were shown in Figure 3. The above limitations were only due to the time of performing iterations. For example, in total about 3 million measurements (simulations) were necessary for the realized change of the number of clusters by one in the range of 1 to 60, at changed frequency of robot’s crossings by every 1% in the range of 10% to 100% of the total area, and 5000 iterations for each of the measured values.

Assuming the surface of the reservoir as the image matrix *L*_*o*_ with a resolution *M*×*N* containing the value "1" in places where the fish occur and "0" in the other ones, the measurement error of the total number of fish in the reservoir is:

(1) $$\delta_{f}=\frac{\sum_{m=1}^{M}\sum_{n=1}^{N}L_{s}\left(m,n\right)}{\sum_{m=1}^{M}\sum_{n=1}^{N}L_{o}\left(m,n\right)}\cdot 100\%$$

where:

(2) $$L_{s}\left(m,n\right)=L_{o}\left(m,n\right)\cdot L_{f}\left(m,n\right)$$

(*m,n*) – are the coordinates of the point (row, column) of the image matrix *L*_*f*_,

*L*_*f*_ – contains the value "1" in the areas covered by the sonar beam and "0" in the other ones.

*L*_*o*_ – has the value "1" in fish locations in the reservoir and "0" in the other ones.

Therefore, the error value *δ*_*f*_ represents the percentage of fish in a flat area of the sonar beam in relation to the number of fish in the reservoir at one depth (the assumption abut a two-dimensional approach is still valid). A discrete image of the reservoir area is not limiting in any way the scope of discussion since the resolution of the images *L*_*f,*_*L*_*o*_ and *L*_*s*_ can be freely expanded. According to this image, the measurements of the impact of changes in the surface percentage measured by sonar *p*_*f*_ and in the number of fish clusters *s* on the value *δ*_*f*_ were carried out. The value *p*_*f*_ was defined as:

(3) $$p_{f}=\frac{\sum_{m=1}^{M}\sum_{n=1}^{N}L_{f}\left(m,n\right)}{M\cdot N}\cdot 100\%$$

**Figure 4:**
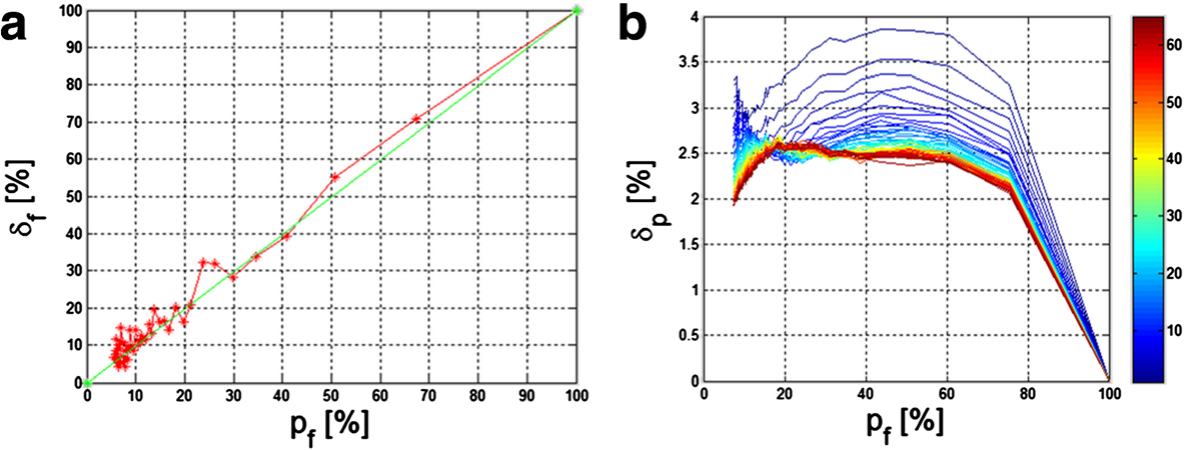
** Changes in the error value *δ***_***p ***_**and *δ***_***f ***_**as a function of percentage changes of the surface covered by sonar.** (**a**) Changes in the value of the measurement error of the number of fish *δ*_*f*_ as a function of percentage changes of the surface covered by sonar (green line - an ideal course, red - simulation result); (**b**) Changes in the error value *δ*_*p*_ as a function of percentage changes of the surface covered by sonar *p*_*f*_ for a variable number of clusters *s* in the range from 1 to 60 (the colors indicate charts for the subsequent number of clusters *s*).

Examples of changes in the value *δ*_*f*_ as a function of changes in the surface percentage measured by sonar *p*_*f*_ were shown in Figure 4.

The linear dependence (Figure 4 a– in red) of the measurement error value of the number of fish *δ*_*f *_as a function of percentage changes in the surface covered by sonar enables to estimate the number of fish based only on the known *p*_*f *_value. The closer the course marked with the red line gets to the ideal diagonal (linear dependence marked with a green line – Figure 4(a), the more accurate calculation of the number of fish is. Therefore, the error *δ*_*p *_ defined as the difference between the green and red courses with the reference to the absolute value, is vital, i.e.:

(4) $$\delta_{p}=\left|\frac{\sum_{m=1}^{M}\sum_{n=1}^{N}L_{f}\left(m,n\right)}{\sum_{m=1}^{M}\sum_{n=1}^{N}L_{o}\left(m,n\right)}-\frac{\sum_{m=1}^{M}\sum_{n=1}^{N}L_{f}\left(m,n\right)}{M\cdot N}\right|\cdot 100=\left|\delta_{f}-p_{f}\right|\;\%$$

**Figure 5:**
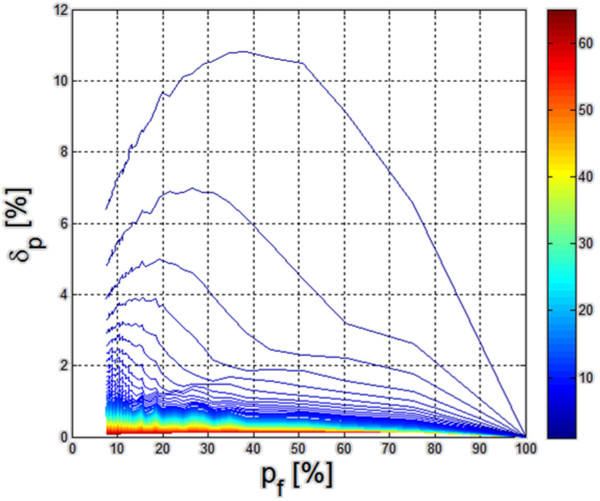
**Changes in the error *δ***_***p ***_**as a function of percentage changes in the surface covered by sonar *p***_***f ***_**for a variable total surface of 10 clusters in the range from 1% to 70% of the whole area.** (The colors indicate charts for the subsequent total surface of clusters).

In the case of a random distribution of fish at a depth of 5 meters covered by the sonar beam with a cone angle of 30°, the simulation results were shown in Figure 4(b). The values of the error *δ*_*p*_ are the highest and exceed 3.8% of a small number of clusters. This is intuitively a correct result – an increased number of clusters reduce the measurement error. The presented graph also suggests the range of values *p*_*f*_ from 10% to 75% in which the error *δ*_*p*_ is not higher than 2.6% of the number of clusters *s* exceeding 40. The impact of both the number of clusters and changes in their surface for individual clusters on the result is automatically interesting. Assuming a change in the total surface *p*_*s*_ of all the clusters (*s* = 10) in the range from 1% to 70% of the whole area, the results obtained are as shown in Figure 5. The graph shown in Figure 5 has several characteristics. The total value of the cluster area exceeding 30% of the entire analyzed area has error values *δ*_*p*_ below 0.5%. The total area of clusters below 10% of the total reservoir surface causes a measurement error exceeding 10%. Therefore, analysis of combined simulation results presented in Figures 4(b) and 5, it was possible to observe range of errors which can be obtained for different degrees of sonar coverage of the reservoir surface by the fish clusters. The maximum value of the error *δ*_*p*_ is 11% and is linked to the *p*_*f*_ value equal to 40% and the degree of covering the reservoir with the fish clusters not higher than 10%. The *p*_*f*_ value below 40% should be selected if the reservoir was considerably covered with fish clusters. For other cases, the error *δ*_*p*_ is equal to a few percent.

**Figure 6:**
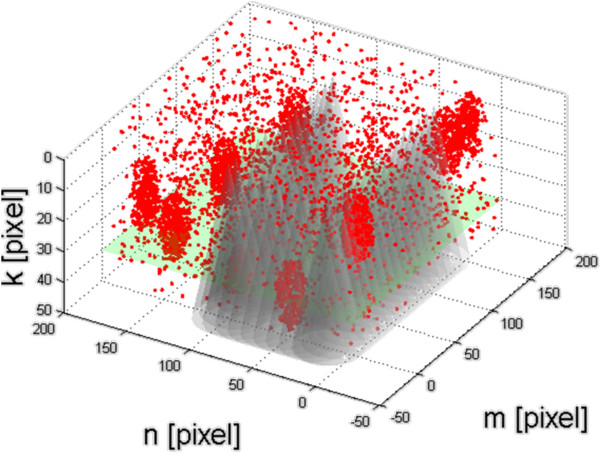
**Random location of the fishes in the coordinates (*m,n,k*) together with 10 clusters which they form (marked in red).** The area covered by the sonar ultrasonic beam cone is shaded in gray. One of the possible discrete positions of the estimation layer was marked in green.

Using simulations for the studied area estimated only for the bottom of the reservoir, considerations were extended to three-dimensional reservoir models and the conical shape of the ultrasound beam. Extending the discussion of the real three-dimensional reservoir and spatially distributed fishes, a discrete model was built and a series of simulations were carried out. Figure 6 shows the graph of random fish located in the coordinates (*m,n,k*) with 10 formed clusters. The area covered by the cone of the sonar ultrasonic beam (an example of discrete location of one of the estimation layer) was also marked. The measurement error *δ*_*ps*_, due to the shape of the ultrasound beam and its incomplete covering of the reservoir, is:

(5) $$\delta_{\mathrm{ps}}=\left|\frac{\sum_{k=1}^{K}\sum_{m=1}^{M}\sum_{n=1}^{N}L_{f}\left(m,n,k\right)}{\sum_{k=1}^{K}\sum_{m=1}^{M}\sum_{n=1}^{N}L_{o}\left(m,n,k\right)}-\frac{\sum_{k=1}^{K}\sum_{m=1}^{M}\sum_{n=1}^{N}L_{f}\left(m,n,k\right)}{M\cdot N\cdot K}\right|\cdot 100\%$$

where:

*k* – discrete depth coordinate,

*K* – maximum depth in pixels.

For the error, defined in this way, a simulation for a random location of fish containing 10 clusters was carried out. The reservoir is magnified with a three-dimensional matrix sized *M*×*N*×*K* where *K* is the total number of levels of depth distinguished by sonar. This is a value strictly dependent on setting the depth scale of the sonar and the resolution of its image. For the used sonar module, it is *K* = 1970 pixels (ping) for each measurement, which, e.g., for a range of 10 meters, means that for one meter there are 197 pixels. As the influence of the number of robot’s crossings on the obtained accuracy *δ*_*ps*_ in the estimation of the number of fish is important, the following assumptions were adopted:

• discrete size of the reservoir – *M*×*N*×*K =* 200×200×50 pixels,

• the number of fish clusters – 10,

• the angle of ultrasonic beam cone of the sonar – 30^o^.

For such assumptions, a simulation was carried out of changes in the error *δ*_*pa*_ defined as:

(6) $$\delta_{\mathrm{pa}}=\frac{\left|\frac{M\cdot N\cdot K\cdot \sum_{k=1}^{K}\sum_{m=1}^{M}\sum_{n=1}^{N}L_{s}\left(m,n,k\right)}{\sum_{k=1}^{K}\sum_{m=1}^{M}\sum_{n=1}^{N}L_{f}\left(m,n,k\right)}-\sum_{k=1}^{K}\sum_{m=1}^{M}\sum_{n=1}^{N}L_{o}\left(m,n,k\right)\right|}{\sum_{k=1}^{K}\sum_{m=1}^{M}\sum_{n=1}^{N}L_{o}\left(m,n,k\right)}\cdot 100\%$$

for a variable number of crossings and thus different *p*_*fs*_ values defined as:

(7) $$p_{\mathrm{fs}}=\frac{\sum_{k=1}^{K}\sum_{m=1}^{M}\sum_{n=1}^{N}L_{f}\left(m,n,k\right)}{M\cdot N\cdot K}\cdot 100\%$$

**Figure 7:**
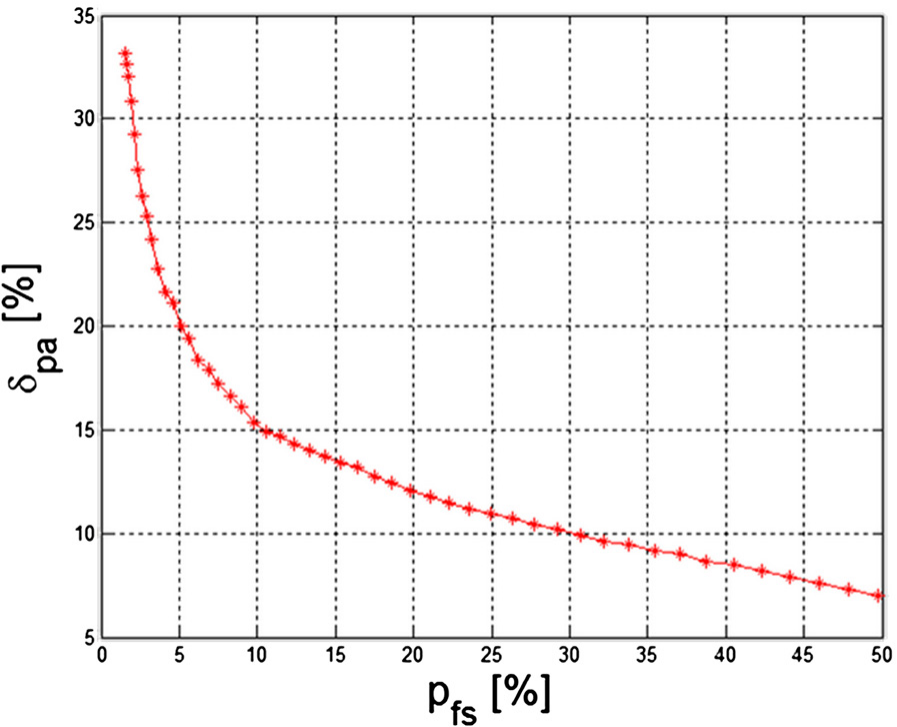
**Changes in the error *δ***_***pa ***_**as the function of percentage changes in the volume *p***_***fs ***_**covered by sonar for a three-dimensional model.** According to the graph shown the values of error *δ*_*pa*_ increase for reduced capacities *p*_*fs*_ analyzed by sonar. This relationship is not linear. As it is apparent from the graph, the value *p*_*fs*_ equal to 10% or 15% is the best choice, taking into account a compromise between the accuracy and coverage of the reservoir volume.

The results are shown in Figure 7. According to the graph shown in Figure 7, the values of error *δ*_*pa*_ increase for reduced capacities *p*_*fs*_ analyzed by sonar. This relationship is not linear. As it is apparent from the graph, the value *p*_*fs*_ equal to 10% or 15% is the best choice, taking into account a compromise between the accuracy and coverage of the reservoir volume. Assuming that the *p*_*fs*_ value is to be 10%, relations between the number of crossings, the cone angle *α* and the depth (number of pixels) *k* of the reservoir were calculated. It can be easily deduced from Figure 3 that:

(8) $$\mathrm{Tm}=9\cdot k\cdot \mathrm{tg}\left(\frac{\alpha }{2}\right)$$

**Figure 8:**
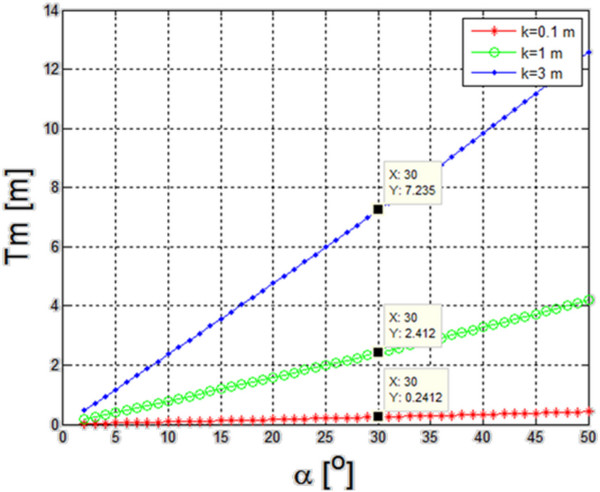
**Changes in the maximum *Tm *value [m] for the value of the angle of the sonar ultrasonic beam cone ranging from 2**^**o **^**to 50**^**o **^**and three different depths of 0.1; 1.0 and 3.0 meters.** Three characteristic points for the angle *α* = 30° are indicated in the graph*.* For such a value of the angle, the maximum distance of neighboring crossings (*Tm*) was 0.24, 2.4 and 7.2 meters for the maximum depths of the reservoir 0.1, 1 and 3 m, respectively. This is very practical information related to the maximum possible distance between adjacent crossings of the robot for a given type of sonar ultrasound head and a given depth.

Using this equation, simulations were carried out of changes in the maximum *Tm* value (expressed in meters), for the cone angle of the sonar ultrasonic beam changes (from 2° to 50°) and three different depths of 0.1, 1 and 3 meters. The results were shown in Figure 8. Three characteristic points for the angle *α* = 30° are indicated in the graph*.* For such a value of the angle, the maximum distance of neighboring crossings (*Tm*) was 0.24, 2.4 and 7.2 meters for the maximum depths of the reservoir 0.1, 1 and 3 m, respectively. This is very practical information related to the maximum possible distance between adjacent crossings of the robot for a given type of sonar ultrasound head and a given depth. In the case of undulated bottom, the mean value of the depth should be taken as the mean value determined on the basis of the data on the bottom depth taken from the sonar.

**Figure 9:**
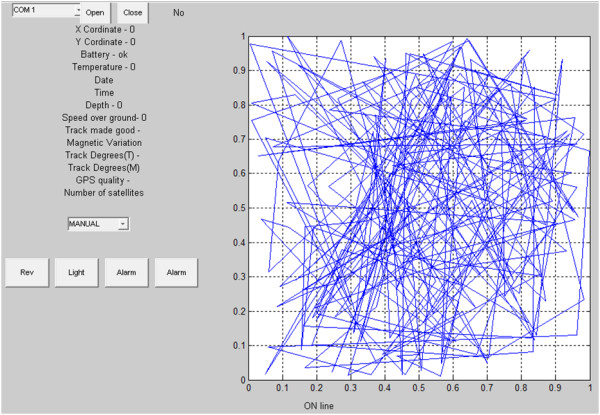
**An example of the software tabs for controlling the robot, data acquisition and analysis.** The tab is dedicated for controlling the robot and monitoring its trajectory. The graph of the trajectory shows random values of location errors resulting from the GPS error (the robot was in a constant location).

The necessary information about the location, depth and physicochemical parameters of the reservoir were transferred wirelessly from the robot to the station. The information about the trajectory sent to the robot from the station was constantly corrected. The software (home tab) for controlling the robot is shown in Figure 9. The software shown in Figure 9 enables to determine the trajectory, manual control of the robot, read the parameters such as depth, GPS coordinates, number of satellites, GPS signal status, battery level, water temperature, date and time on the UTM, the compass data and information about straying of the robot from the designated path.

The analyses identified many factors which might have an effect on the error related to measurements of the number of fish. However, they can be easily reduced and their impact on the result can be estimated. The presented methodology involves the use of images acquired from the sonar as one of the main (besides location) sources of the information on the number of fish. The authors used here already available and own new methods of image (from sonar) analysis and processing.

### Analysis and processing of data from the sonar

The data analysis from the sonar concerned automatic recognition of fish using the methods of image analysis and processing. Input image *Lwe* with a resolution of *M*×*N* = 1970×200 underwent subsequent stages of analysis [14-16], i.e.:

• filtration with a median filter whose mask size is 3×3 which enables to eliminate small artifacts and noise,

• morphological opening for a large structural element (40×40 pixels) which enables to remove uneven brightness of particular pixels,

• binarization of the resulting image with the threshold being 20% of the total average brightness in the analyzed image,

• labeling of individual fish which enables to count them and calculate what area they occupy.

**Figure 10:**
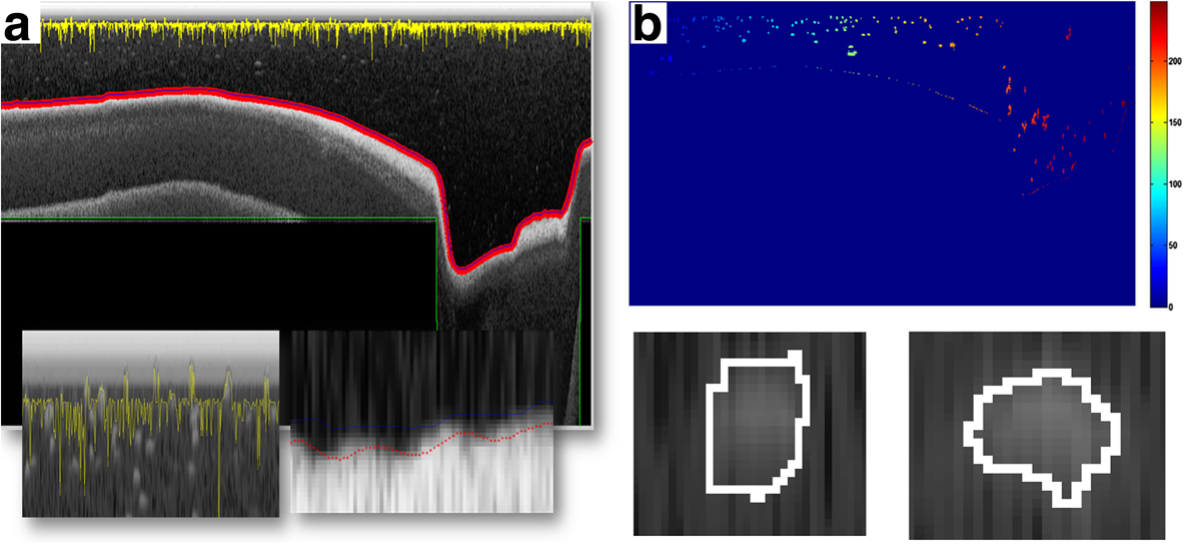
**Sample image from the sonar and its processing.** (**a**) marked elements of the imge (red and blue – “Depth”, yellow – “SurfaceValid”, green – “DepthLimit”); (**b**) The image of labels (each fish gets its own label) and problems with marking and evaluation of the correctness the outer edge of the fish contour.

All these parameters were adjusted once for a given type of sonar module during the production stage. The user and the operating person introduce no parameters for both, the software and robot settings. Figure 10(a) showed the example of an image from the sonar, and the result of its processing in accordance with the presented methodology. As is apparent from the analysis of Figure 10, problems with the automatic marking of the fish location using the described method relate to the separation of images of closely adjacent fish. In this case, the separation can only be based on the knowledge about fish species and their size in the reservoir. The methods of image analysis and processing fail at this point, because there is no known pattern related to the searched shape. Moreover, fish contours are not distinguishable in places where they overlap. There are known methods of separation of linked objects but they cannot be used for splitting a formless object. Fish location (GPS coordinates) and the depth at which it was found are known from the above analysis of the received data. The center of gravity of the fish is taken as a determinant. Worth mentioning is that information about the GPS coordinates are not listed in each ping of information coming from the sonar. This is due to the specificity of operation of both the sonar module and GPS module. The sonar module generates the ultrasonic beam several times per second (or dozens of times) while the GPS module does it only once or twice per second.

### Verification of the adopted measurement method

**Figure 11:**
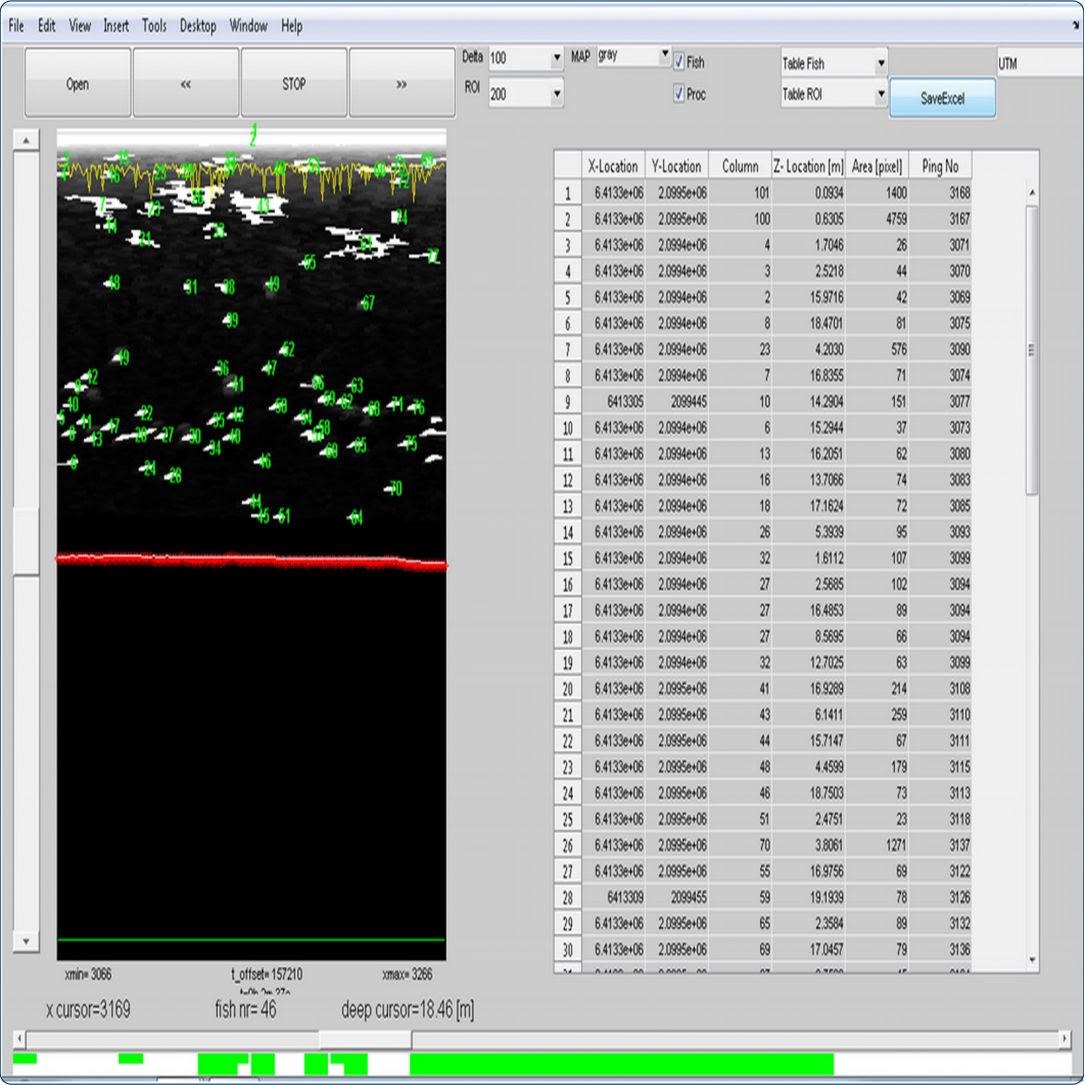
**Software tab for the acquisition and calculation of the number of fish in the reservoir.** The window on the left,shows the images of counted individual fish (green), the bottom line is marked in red, and the water surface in yellow. The table on the right, shows the arrengements of the database with records about the location and size of the identified fish.

Verification of the effectiveness of the robot and the method of measuring the number of fishes was based on the created software tested in Matlab and then rewritten to the C++. The main window of the program consists of two parts: The results of automatic recognition and indexing of fish are seen on the left window. The right side contains the table with the information on the location, volume and the number of the recognized object (fish) in successive rows. Buttons at the top of the window enable to navigate the saved image coming from the sonar and change the visible area of analysis. Moreover, it is possible to hide or expose the selected fragment of analysis. The belt at the bottom of the window shows in green and white the studied areas and inform whether the GPS coordinates were sent. In case of very bad weather, a correct image from the sonar can be archived but the GPS coordinates will not be saved. Then it is possible to identify individual fish, but this information cannot be applied to their location in the reservoir (Figure 11).

The relationships shown in Figure 8 were verified experimentally. The number of fish was estimated in three typical breeding ponds:

• reservoir *A*, 56 m × 26 m × 1.5 m (length × width × depth),

• reservoir *B*, 55 m × 26 m×1.5 m,

• reservoir *C*, 130 m × 22 m × 1.3 m.

Sonar measurements were carried out for each reservoir, and then all the fish from the ponds were caught and underwent a direct quantitative and qualitative analysis. A list of fish species, their mean body mass and body length was given in Table 1.

**Table 1:** Data on fishes collected in three reference fish ponds

***No***	**Day of sampling**	**The pond**	**Fish species**	***R***	**Total body mass (kg)**	**Mean body mass (kg)**	**Body length (range in cm)**	Mean* body length (in cm)
1	15.10.2012	*A*	*Pseudorasbora parva*	172	0.86	0.003	4.5 - 8	5.5
2	15.10.2012	*A*	*Perca fluviatilis*	200	8.5	0.042	14 - 17	15.9
3	15.10.2012	*A*	*Cyprinus carpio* (2 years old)	100	33.1	0.331	22 - 32	26.7
4	15.10.2012	*A*	*Cyprinus carpio* (3 years old)	56	98	1.75	38 - 50	45.2
5	15.10.2012	*A*	*Leuciscus cephalus*	17	1	0.059	12 - 21	15.5
6	15.10.2012	*A*	*Carassius gibelin*	1	0.05	0.05	11	-
7	15.10.2012	*A*	*Rutilus rutilus*	2	0.15	0.075	10 - 12	-
8	15.10.2012	*B*	*Cyprinus carpio* (2 years old)	171	217.2	1.27	35 - 47	40.1
9	17.10.2012	*C*	*Cyprinus carpio* (4 years old)	120	341	2.84	45 - 67	-
10	17.10.2012	*C*	*Cyprinus carpio* (3 years old)	84	110	1.31	40 - 49	-
11	17.10.2012	*C*	*Ctenopharyngodon idella*	2	5	2.5	68 - 71	-
12	17.10.2012	*C*	*Esox lucius*	19	22	1.2	48 - 53	-
13	17.10.2012	*C*	*Sander lucioperca*	1	0.8	0.8	38	-
14	17.10.2012	*C*	*(Leuciscus leuciscus, Pseudorasbora parva, Perca fluviatilis, Rutilus rutilus)*	-	12	-	**4 - 17**	-

*Only in case when at least 10 specimens were caught; *R*– number of specimens.

The robot automatically followed the set trajectory. The automatic trajectory correction was possible to a limited extent because of the wind. Keeping the trajectory for allowing only slight variations needs more energy for the driving motors and reduce the time available for continuous robot operation.

The results of direct counting were used as the reference values for the determination of the error of the described measurement method. Using the collected data, the total number of fish in the reservoirs were calculated (Table 2). A comparison of the total number of counting (Table 2) demonstrated validity of the results obtained from the simulation. For the reservoir *A*, the measurement error was of 8% and for the reservoirs *B* and *C*, it was 7% and 8%, respectively. These values are within the acceptable error range of the percentage volume analyzed by the sonar in relation to the total volume of the reservoir. This confirms the usefulness of the robot for estimating the number of fish in the reservoir.

**Table 2:** Total number of fish using catches (reference value) and the robot

**Couting method/Name of the pond**	***A***	***B***	***C***
Number of fish – routine sampling	548	171	226
Number of fish – sonar counting	502	182	245
The relative volume of the reservoir analyzed with sonar	11.5%	9.6%	12.5%

Symbols for particular ponds correspond to the Table 1.

## Discussion

### Selection of tools for the quantitative estimation of fish in the reservoir

A direct analysis of the number of fish is performed using sonar attached to the boat. Commercial software for most types of sonar enables automatic marking of fish and their location. However, it is not possible to approximate the results obtained for the whole water reservoir. Various types of robots, mainly for monitoring the water bodies, are also used. These include Robo-fish developed by the LiveScience in partnership with the National Science Foundation [17]. The inventors of the fish-like robot [17] stated that “the hydrodynamic shape minimizes drag and with this shape the robot fish can move through the water using rhythmic body and fin motions. Such movement offers much better maneuverability than propeller-based propulsion, allowing the robots to, for example, turn within a tight radius. That kind of maneuverability is especially helpful in dealing with the turbulences and currents the robots often encounter”. This product could be used as a hybrid serving as the fish-robot and underwater glider and is rather devoted to large and deep water areas. Another fish-shape, 1.5 m long robot, has been recently created under the EU project by a group of scientists from the University of Essex and the University of Strathclyde, UK, Ireland's Tyndall National Institute and Thales Safar [18]. The robot was produced in order to reduce time of detecting pollutants (for example oil spills) in large water bodies “from weeks to seconds” monitoring divers and rescue at sea. Chemical sensors fitted to the robot-fish could collect data of specific pollutants leaking from the ships or the bottom pipelines. The robot swim independently and could map the actual position and transmit the data to the base. The robot also know how to return safely to the base when the battery life is running slow. Unfortunately, this product is highly expensive (ca. 20’000 pounds) and could not be used in small water bodies with low transparency.

Our model differs from the above mentioned robots in terms of details related to the wireless transmission transmission of the data. The device presented here, allows encrypted transmission of the data and automatic calculaton of the number of fishes. The operation with this model is autonomic – our robot automatically returns to the station when a voltage drop is detected.

Various types of the wired robots are also available. For example, Hayes et al. [19], described analyses done by a cylindrical, 1.3 m long robot containing separated transmitter and receiver of the sonar supported with the GPS module and NMEA transmission. However, the use of wired connection significantly limited the range of applications. This equipment may be sufficient for large and deep reservoirs. In shallow reservoirs, such as the aquaculture water bodies or shallow lakes, the use of robots of that type would be problematic.

### Methods of the assessment and quality of the estimation fish number and biomass

The estimation of the number of fish in the reservoirs based on the data derived from the sonar was presented in a series of publications [20-28]. Taking into account criteria of the technical construction and cost of the product, one of the most important elements. seems to be the type of the used transducer Omni directional type multi-beam enables full reconstruction of the location of fish and the scanned area in all directions [29-33]. In the case of full reconstruction of 3D images this model allows to estimate the number of fish with almost 100% accuracy. One-beam type of head does not give such accuracy. For example, Maxwell and Gove [34] compared calculations for the number of salmon migrating to the spawning grounds for the Bendix and DIDSON sonars. Using linear regression they found that the errors ranged within several tens of percent. Analytic methodology for measuring tthe number of fish are carefully described when the fish biomass is estimated. Tuser et al. [35] showed that for sloped areas the underestimation of fish density ranged from 21% to 39% in abundance and from 5% to 12% in biomass.

Quantitative analyses using split-beam are more convenient in in the assessment of large rivers Matveev [36] obtained satisfactory results for the fish sized from 18 to 150 mm. It is definitely more difficult in small and shallow reservoirs. Another major problem in the sonar operation is the acoustic zone, but the research in this area was carried out mainly in the benthic zone of the sea, [37] and [38]. One possible solution in such conditions is mounting a sonar transducer from the bottom side [39].

In most of the analyzed studies, the authors recommend calibration of the adopted method to specific conditions of the measurement. Then it is possible to obtain quite accurate measurements with an error as low as 3% [40]. In the present study, the accuracy obtained for the robot,was dependent on the measurement conditions. Comparisons with the reference direct catching of fish showed 12.5% error estimation for sonar measurements. The mobile sailing robot presented in the paper may be used in the automatic monitoring of water quality and quantitative fish estimation. Despite a higher measurement error, some imperfections indicated in this study, should be eliminated in future models developed for the mass production. These include:

• low impact resistance – despite the hull made of 5 layers of the glass fiber, it is little resistant to shocks during transport,

• low resistance to high waves – despite the proper balance of the robot, larger waves can cause swinging of the boat, which results in the appearance of artifacts in sonar images,

• selection of batteries, that enable evaluation of the capacity in case of their partial wear or damage,

• cylindrical arrangement of solar panels, in order to reduce compliance to dirt (feces of birds, algae, etc.),

• sending the GPS data for each sonar ping – used GPS module sent data every 10–15 sonar pings,

• lack of automatic calibration of complex probes,

• relatively high price of the production (2'500 Euros without specialized probes). The authors intend to modify the used modules and to develop a more cost-effective model.

The developed software works correctly in the continuous operation of the robot for up to several hundred hours. Each loss of the wireless contact starts a self-test procedure. The robot can work continuously (without charging and return to the base) for 3 hours. Depending on the weather conditions, it can perform measurements over a distance of several kilometers.

## Concluding remarks

The created robot together with the developed software meets all the necessary assumptions. The listed above disadvantages, can be eliminated in further work of the project. The robot works properly in a variety of harsh weather and environmental conditions, is fully automated and can be remotely controlled using Internet. The main advantage of the robot is the ability to monitor the reservoirs that supply drinking water. This is very important for human health. It enables to obtain the said parameters from any selected area of a given reservoir and in any period of time. Moreover, the robot does not require any participation of the operating personnel. All the actions (control of the robot) and transfer of the results can be done remotely from different places in the world. Alternatively, the practical usefulness for the protection of human health is the analysis of the areas of water bodies used as public swimming pools. The advantage of the robot is the possibility of its use for the quantitative analysis of fish in small, shallow inland water bodies.

## Abbreviations

GPS: Global Positioning System; MAC: Medium Access Control; UTM: Universal Time Clock; NMEA: National Marine Electronics Association.

## Competing interests

The authors declare that they have no competing interests.

## Authors’ contributions

RK and ZW suggested the algorithm for image analysis and processing, implemented it and analysed the images. AW and BŁ formulated the concept of connections low-cost sonar with remote mini-boat to estimate fish populations in small reservoirs. SW, AK, AW, BŁ, MP, JK, PM performed the acquisition of the sonar images and consulted the obtained results. All authors have read and approved the final manuscript.
